# Methods for Assessment of Sleep and Circadian Rhythms in Cardiovascular Research

**DOI:** 10.1007/s11906-025-01345-4

**Published:** 2025-10-21

**Authors:** Rebecca Williams, Gabrielle Gloston, Katherine C. Ward, Shubhi Jain, Kristen Hays, Annie Ensor, Samarth Patel, Neel Patel, Mackenzie Hogue, S. Justin Thomas, Brittanny M. Polanka

**Affiliations:** 1 Department of Psychology, University of Alabama at Birmingham, Birmingham, AL United States of America; 2 Department of Psychiatry & Behavioral Neurobiology, University of Alabama at Birmingham, Birmingham, AL United States of America; 3 Department of Neurobiology, University of Alabama at Birmingham, Birmingham, AL United States of America; 4 Department of Clinical and Diagnostic Sciences, University of Alabama at Birmingham, Birmingham, AL United States of America; 5 Division of General Internal Medicine and Population Science, University of Alabama at Birmingham, Birmingham, AL United States of America

**Keywords:** Sleep, Circadian rhythm, Sleep measurement, Circadian biomarker, Cardiovascular

## Abstract

Sleep is recognized in Life’s Essential 8™ as an important behavioral factor for improving and maintaining cardiovascular health. While sleep duration is currently the focus in Life’s Essential 8™, sleep health is multidimensional and encompasses regularity, satisfaction, next-day alertness, timing, efficiency, and duration. In addition to sleep, circadian factors have also been implicated in cardiovascular health. For example, shift work, which results in significant circadian misalignment, is associated with poor cardiovascular health. This review will describe methods for measuring, analyzing, and interpreting sleep and circadian rhythms in humans. Subjective and objective measurements of sleep are not always concordant and may reflect distinct constructs. Therefore, both subjective and objective sleep measurements are discussed. Assessment of the circadian system in humans typically relies on the measurement of circadian biomarkers (i.e., melatonin, core body temperature, and/or cortisol) during rigorous and burdensome research protocols. However, novel approaches are being developed to estimate circadian parameters with lower cost and participant burden. This review aims to inform cardiovascular scientists and clinicians of common practices in the assessment of sleep and circadian rhythms.

## Introduction

Interest in sleep and circadian measures is growing in the cardiovascular field. This interest is the result of a large and expanding literature implicating sleep and circadian factors in the development of multiple cardiometabolic risk factors and a subsequent elevation in cardiovascular disease (CVD) event risk [1, 2]. In response, the American Heart Association updated their cardiovascular health metric to the Life’s Essential 8™, including sleep duration as a key behavioral factor for maintaining optimal cardiovascular health [3]. This was a major step forward in furthering public knowledge and awareness of the cardiotoxic consequences of suboptimal sleep. However, sleep is a multidimensional construct that has complex interactions with the circadian system influencing cardiovascular health, which is not captured through the measurement of a single parameter.

Multidimensional sleep health [4] encompasses sleep regularity, satisfaction, next-day alertness, sleep timing, and sleep efficiency (SE) in addition to sleep duration. Sleep can be further conceptualized or examined through the characterization of (a) the components of a sleep window (e.g., sleep onset latency [SOL], wake after sleep onset [WASO]), (b) behaviors that affect sleep (e.g., sleep hygiene), and/or (c) diagnostic criteria or symptoms of sleep disorders (e.g., insomnia). The American Heart Association has recently recognized the importance of sleep health as a multifaceted contributor to cardiovascular health, calling for future cardiovascular research to include multiple metrics of sleep beyond solely sleep duration [1]. A considerable proportion of existing sleep research has taken a limited, unidimensional approach to sleep, often measuring only one sleep dimension with sleep duration and presence of obstructive sleep apnea (OSA) among the most common. A multidimensional approach to sleep research should be emphasized. When choosing sleep measures, it is crucial to consider the differences between—and benefits of using—both subjective and objective sleep measures. Sleep factors can be measured through subjective self-reported questionnaires and through objective assessment via actigraphy or polysomnography. Subjective and objective sleep likely reflect complementary but distinct constructs, and ideally, both should be considered in study design development.

The circadian system aids in regulating sleep-wake cycles. The “two-process model of sleep regulation” posits that sleep is co-regulated by: the circadian pacemaker and the homeostatic process [5, 6]. The circadian pacemaker describes the endogenous circadian rhythms produced by the central and peripheral clocks that produce a roughly 24-hour rhythm. The central clock resides in the suprachiasmatic nucleus of the hypothalamus and is primarily entrained by light. The peripheral clocks reside in various tissues throughout the body and receive input from both the central clock and exogenous cues (e.g., sleep timing, meal timing, and physical activity) [7].

While the primary circadian pacemaker is located in the suprachiasmatic nucleus, circadian clock molecular machinery can be found in almost all cells throughout the body. The circadian clock operates as a transcriptional-translational feedback loop consisting of core components BMAL, PERIOD (PER), CRYPTOCHROME (CRY), and CLOCK. CLOCK and BMAL1 heterodimerize and activate transcription of *Per* and *Cry* genes. Eventually, PER and CRY protein products inhibit CLOCK and BMAL activity, thus closing the loop over a roughly 24-hour cycle [8, 9]. Some clock genes (i.e., Per2, Per3, Cry2, and Cry1) have been implicated in familial forms of Advanced and Delayed Sleep-Wake Phase Disorders [10–13].

The homeostatic process describes the sleep-wake dependent drive to sleep, which increases as the duration of wakefulness increases [5, 6]. Complex and not-fully-understood interactions between these processes regulate and influence a person’s physiological and behavioral cues for sleep-wake cycles (as well as other circadian cycles, e.g., hunger). Measurement of the circadian system relies on objective measurement of circadian biomarkers under controlled conditions. Given the wealth of potential measurement approaches and variables across sleep and circadian factors, the needs of a given research study and its hypotheses should drive the design and variable selection.

It has become clearer in recent years that sleep—both its many facets and as an aggregate—is crucial to understanding changes in blood pressure (BP) and predicting hypertension risk [14–17]. Thus, the goal of the present review is to support the increasing interest at the intersection of sleep, circadian rhythms, and cardiovascular research by equipping cardiovascular researchers with recommendations for methods and statistical analyses used in sleep and circadian rhythms research. We summarize common assessment and analytic approaches to accurately measure, analyze, and interpret sleep and circadian data to advance their inclusion in cardiovascular research.

## Methods

### Semi-Structured Clinical Interviews

One commonly used interview is the Structured Clinical Interview for Sleep Disorders-Revised (SCISD-R). This is designed for trained health professionals familiar with the Diagnostic and Statistical Manual V (DSM-5) to screen for sleep disorders. It covers insomnia, hypersomnolence, circadian rhythm sleep-wake, nightmare, rapid eye movement sleep arousal, and rapid eye movement sleep behavior disorders, as well as OSA, restless legs syndrome (RLS), and narcolepsy [18, 19]. It is useful for standardization in research studies to identify presence of possible sleep disorders, as well as for diagnostic clarification in behavioral sleep medicine settings. The SCISD-R can be especially beneficial when questionnaire data is inconclusive, or when sleep complaints are complex. The SCISD-R is the only widely used semi-structured clinical interview dedicated exclusively to sleep disorders, though other notable mentions include the sleep module of the Mini International Neuropsychiatric Interview [20] and the sleep disorders section of the Structured Clinical Interview for DSM-5 [18].

### Subjective Sleep Measures

Subjective sleep assessment includes self-report prospective sleep diaries and retrospective questionnaires. Sleep diaries and questionnaires are among the most cost-effective sleep assessments [21]. Most sleep disorders (OSA being a notable exception) can be diagnosed based on patient report. Thus, subjective sleep measures play a critical role in tracking symptomatology in both clinical and research settings. This section will describe the use of sleep diaries and the most frequently used questionnaires in research for measuring sleep quality, chronotype, and sleep disorder symptomatology. Clinically oriented measures are included for the consideration of researchers given that the goal of some studies may require identification and recruitment of participants with clinical diagnoses or selection of a clinically important patient outcome measure in a behavioral or pharmacological intervention clinical trial study (e.g., ensuring the successful treatment of insomnia in order to examine subsequent benefits on cardiovascular health metrics). Both subjective and objective sleep measures are summarized in Table 1.

**Table 1:** Summary of included subjective and objective sleep measures

	Assessment	Domains Measured	Pros	Cons
Subjective Sleep Measures				
Sleep Diaries	~ 8 items Timeframe: 7 days	Time into bed, time attempt to fall asleep, time awake, time out of bed, number of awakenings, SOL, WASO, TST, TIB, SE	Prospective Standardized form available Used in clinical practice May capture circadian rhythm patterns	Not all are standardized
Pittsburgh Sleep Quality Index	19 items Timeframe: past month	Global Sleep Quality Sub-categories: sleep quality, SOL, TST, habitual SE, sleep disturbances, use of sleep medication, daytime dysfunction	Most widely-used scale for sleep disturbance Measures global and sub-categories of sleep domains	Retrospective Only captures past month Not for clinical diagnosis No sleep disorder specificity
RU-SATED	5 items Timeframe: generally	Sleep regularity, sleep satisfaction, daytime alertness, sleep timing, SE, duration	Multidimensional, positively framed	Not for clinical diagnosis or screening
Sleep Hygiene Index	13 items Timeframe: generally	Sleep hygiene practices	Good for assessing sleep hygiene practices	Insufficient for understanding and modifying sleep behaviors
PROMIS Sleep Disturbance Short Form	8 items Timeframe: 7 days	Sleep restlessness, satisfaction, quality, TST, SOL, WASO	Standardized scoring Predictive validity Validated against longer forms Adult, pediatric, and parent proxy versions available	No sleep disorder specificity
PROMIS Sleep-Related Impairment Short Form	8 items Timeframe: 7 days	Daytime dysfunction due to poor sleep	Standardized scoring Adult, pediatric, and parent proxy versions available	No sleep disorder specificity
Morningness-Eveningness Questionnaire	19 items	Preferred time of day for peak alertness	Correlated with DLMO Based on preference rather than behaviors for circadian assessment	Potential for geographic bias
Munich Chronotype Questionnaire	17 items	Infers chronotype from behavior: work schedule, workday sleep schedule, free day sleep schedule, self-assessment of chronotype	Nuance of free work vs. free days Adjustment for social jet leg	Cannot be used for those with irregular work schedules
Ultra-Short Munich Chronotype Questionnaire	6 items	Infers chronotype from workday and free day sleep schedule; self-assessment of chronotype	Brief Highly correlated with full version	Less comprehensive; does not capture light exposure, alarm clock use, sleep inertia
Insomnia Severity Index	7 items Timeframe: last 2 weeks	Severity of insomnia symptoms, sleep satisfaction, daytime dysfunction, concern for sleep problems	Widely used Aligns with DSM and ICSD diagnostic criteria	Screening tool, not a diagnostic tool Does not capture all aspects of insomnia, like EMAs
STOP Bang Questionnaire	8 items	OSA symptoms and features	Affordable screener Validated against PSG	Requires measurement of neck circumference
Berlin Questionnaire	11 items	OSA symptoms and features	Affordable screener	Low positive predictive value
Cambridge Hopkins Diagnostic Questionnaire	11 items	Features of RLS and similar non-RLS features used in differential diagnosis	Includes differential diagnosis questions	Potential for misidentification of conditions that mimic RLS Face-to-face format not psychometrically tested
International Restless Legs Syndrome Rating Scale	10 items Timeframe: past week	Severity of RLS symptoms among already diagnosed RLS patients	Widely used Comprehensive RLS assessment	Not recommended for screening
Epworth Sleepiness Scale	8 items Timeframe: Current	Situational daytime sleepiness for specific activities	Widely used	Pre-specified activities to be rated
Karolinska Sleepiness Scale	1 item Timeframe: prior 10 min	Situational daytime sleepiness at present 9-point scale to be used in rating	Sensitive to change Correlated with EEG and behavioral indicators of sleepiness Researcher can choose activities to be rated	Limited scope (only prior 10 min) Limited sample size in validation studies
Stanford Sleepiness Scale	1 item Timeframe: Current	Situational daytime sleepiness at present 7-point scale to be used in rating	Sensitive to change Researcher can choose activities to be rated	Limited scope (only current) Limited sample size in validation studies
Functional Outcomes of Sleep Questionnaire	30 items (long), 10 items (short) Timeframe: generally	effects of fatigue on daytime activities and quality of life	Comprehensive High validity and reliability	Length of full version
Dysfunctional Beliefs and Attitudes about Sleep Scale	30 items (long), 16 items (short) Timeframe: generally	Sleep-related beliefs and cognitions that can perpetuate sleep disturbance	High reliability and validity Useful in clinical settings	Length of both versions Does not capture behavioral factors
Objective Sleep Measures				
Actigraphy	Recommended timeframe: 7–14 days	SOL, WASO, TST, TIB, SE Variability/irregularity in these indices	Validated against PSG Non-invasive Well-tolerated In-home sleep measurement Measurement of light exposure (on some devices) May identify circadian rhythm patterns	Not used clinically Measurement is based on movement Need sleep diary to inform software scoring
Polysomnography	Recommended timeframe: at least 2 nights	Montages: EEG, EOG, EMG, ECG, nasal pressure, respiratory effort, pulse oximetry. Sleep stages (N1, N2, N3, REM), SOL, WASO, TST, TIB, SE, number of arousals, AHI, oxygen saturation,	Used to diagnosis SDB, periodic limb movement disorder, and parasomnias Only available assessment to stage sleep Ambulatory PSGs available	Expensive and invasive May affect generalizability if done in sleep lab

*AHI A*pnea hypopnea index, *DLMO D*im light melatonin onset, *DSM* Diagnostic and statistical manual, *ECG* Electrocardiography, *EEG* Electroencephalography, *EMG* Electromyography, *EOG* Electrooculography, *ICSD* International classification of sleep disorders, *OSA* Obstructive sleep apnea, *PSG* Polysomnography, *RLS* Restless leg syndrome, *SDB* Sleep disordered breathing, *SE* Sleep efficiency, *SOL* Sleep onset latency, *TIB* Time in bed, *TSP* Total sleep time, *WASO* Wake after sleep onset, *EMA* Early morning awakening

### Sleep Diaries

Sleep diaries are short, post-sleep questionnaires that are completed for at least seven consecutive days [22]. Typical questions include estimates for: time spent in bed, number of hours slept, time it took to fall asleep, number of nighttime awakenings, wake time, and activities performed the previous day that may have impacted sleep (e.g., medication, alcohol, caffeine, exercise) [22]. From sleep diaries, it is possible to calculate the following components of the sleep window: SOL, WASO, total sleep time (TST), total time in bed (TIB), and SE (which is a percentage calculated by dividing TST by TIB and then multiplying by 100). Most sleep diaries also have a Likert rating of sleep quality or satisfaction [23]. Sleep diaries are available in both physical and electronic (web- and application-based) formats, with electronic sleep diaries having the capacity to automate scoring, document the day and time a sleep diary is completed, and help avoid “parking lot syndrome,” which is when a person retrospectively fills out their diary with multiple nights of sleep at once [24].

The Consensus Sleep Diary was created to help facilitate consistency of the data being collected from sleep diaries in both clinical and research settings [23]. There are nine items on the core Consensus Sleep Diary: (1) time the respondent got into bed; (2) time the respondent attempted to fall asleep; (3) SOL; (4) number of awakenings; (5) duration of awakenings; (6) time the respondent awoke for the final time; (7) time the respondent left their bed; (8) perceived sleep quality; and (9) an additional space for open-ended comments [23]. There is also an extended Consensus Sleep Diary and a pediatric Consensus Sleep Diary. Sleep diaries are especially useful because of their capacity to detect multiple sleep factors, highlighting their utility in studies interested in multidimensional sleep health. For example, from a Consensus Sleep Diary completed over multiple weeks, one can extrapolate patterns of stability or irregularity in sleep duration, bed timing, wake timing, and mid-sleep timing.

### Sleep Quality Questionnaires

#### Pittsburgh Sleep Quality Index

The Pittsburgh Sleep Quality Index (PSQI), one of the most frequently used subjective measures of sleep quality [19], was designed to measure patients’ global sleep quality and sleep disturbances over the past month. The PSQI is not specific to any one sleep disorder; rather, it captures sleep quality regardless of sleep disorder status. The PSQI is a reliable method of differentiating between “good” and “bad” sleepers [25]. There are 19 self-reported questions and five optional bed partner questions that cover seven components [26]. These components include subjective sleep quality, SOL, TST, habitual SE, sleep disturbances, use of sleep medication, and daytime dysfunction [25]. The total score ranges from zero to 21, with higher scores reflecting poorer sleep quality [26]. A global score higher than five indicates a “poor” sleeper [27]. Sub-scores of the PSQI can be calculated for individual components of sleep quality (e.g., TST, SOL, SE, daytime dysfunction) [28]. Like sleep diaries, the PSQI also captures sleep timing (i.e., bed timing, wake timing, and sleep onset latency), though it does not offer insights into the regularity of that sleep timing over the 1-month period being queried.

#### RU-SATED

The Satisfaction, Alertness, Timing, Efficiency, and Duration (SATED) Scale is designed to measure these components within a multi-dimensional sleep health framework. SATED approaches sleep health with a positive reference frame and emphasizes the positive impact of good sleep on overall health [4]. The SATED has been modified to include sleep regularity and is now called the RU-SATED. The RU-SATED includes the following statements measured on a Likert scale: (1) I go to sleep and wake up at about the same time every day, (2) I sleep 7–9 h per night, (3) The middle of my sleep period is between 2:00 am and 4:00 am, (4) I am awake for less than 30 min between the time I go to bed and the time I get out of bed, and (5) I stay awake all day without dozing, and (6) I am satisfied with my sleep. This measure is psychometrically valid [29].

#### Sleep Hygiene Index

Sleep hygiene refers to the practices and behaviors that produce the best environment for quality sleep to occur. Sleep hygiene is strongly related to sleep quality and modestly related to daytime sleepiness [30, 31]. Some common sleep hygiene tips include maintaining a regular sleep and wake schedule (even on the weekends), avoiding stimulants like caffeine and nicotine, and establishing a sleep routine. The Sleep Hygiene Index is a 13-item self-report questionnaire that quantifies a person’s current sleep hygiene practices. The Sleep Hygiene Index has a good internal reliability (Cronbach’s α = 0.66) and a high test-retest reliability (*r*(139) = 0.71, *p* < 0.01). It is also highly correlated with the PSQI (described above) and the Epworth Sleepiness Scale (ESS; described below) [30].

#### Circadian Chronotype Questionnaires

Chronotype is a circadian-driven preference for certain bedtime and waketimes and is typically categorized as morningness, intermediate, or eveningness. Chronotype reflects someone’s natural preference for when the person feels most awake, sleepiest, and what times a person prefers to sleep. There are specific questionnaires designed to assess chronotype [22].

#### Morningness-Eveningness Questionnaire

The Morningness-Eveningness Questionnaire (MEQ) is used to identify one’s preferred time of day to complete various daily activities and consists of 19 questions, using both Likert and time scales [22]. The scores range from 16 to 86: 16–30 is definitely evening type, 31–41 is moderately evening type, 42–58 is intermediate type, 59–69 is moderately morning type, and 70–86 is definitely morning type. This questionnaire was developed by researchers James A. Horne and Olov Östberg in the 1970s and was first validated using participants between 18 and 32 years old [32]. The full-scale internal consistency is 0.82 [32]. The MEQ is highly correlated (*r* = −0.70, *p* < 0.001) with dim light melatonin onset (DLMO), the gold standard objective method for determining circadian phase [33]. The MEQ has also been validated for children and adolescents [34, 35], and has been translated for several languages [36–38].

#### Munich Chronotype Questionnaire

The Munich Chronotype Questionnaire (MCTQ) assesses chronotype using the sleep midpoint on free or non-workdays. Sleep timing on non-workdays is highly influenced by the individual’s circadian clock, and could help identify chronotype [22]. The MCTQ contains 17 items and four distinct categories, including work schedule, workday sleep schedule, free day sleep schedule, and self-assessment of chronotype [39]. The questions gather information about sleep times, fully awake times, SOL, and sleep inertia (i.e., temporarily reduced cognitive and sensory-motor function that can occur shortly after waking) [40]. A study comparing the validity of the MCTQ with wrist actigraphy found a strong correlation between the two measurements with an *r* value of 0.73 and a *p*-value of < 0.001 [41]. The MCTQ has also been modified for shift workers (MCTQShift) due to the additional difficulties shift workers often experience with sleep [22, 42]. There is also an ultra-short version of the MCTQ, the µMCTQ, which reduces it from 17 items to 6, and has been externally validated with DLMO [43].

It is worth emphasizing the distinction in chronotype assessment between the MEQ and MCTQ. The MEQ utilizes personal preference whereas the MCTQ utilizes sleep behavior (i.e., sleep midpoint) on free or non-workdays. Theoretically, sleep on free or non-workdays is sleep closer to an individual’s preferred time. However, due to schedule constraints and life circumstance (e.g., childcare of young children), this may not always be the case. Researchers will need to consider their population of interest when choosing between these measures. However, we caution researchers from using a sleep diary or portions of the PSQI on sleep timing to infer a person’s chronotype. Researchers are instead recommended to include a validated measure specific to chronotype such as the MEQ or MCTQ. If a researcher is interested in proxies of circadian misalignment, then the use of a sleep diary in conjunction with a circadian questionnaire like the MEQ may provide some insight into how aligned or misaligned a person’s sleep is compared to what their preferred (i.e., circadian preference) sleep would be.

#### Clinical Sleep Disorder Questionnaires

Insomnia is one of the most studied sleep disorders due to its impact. An estimated one third of U.S. adults report insomnia symptoms, with 4–26% meeting clinical diagnostic criteria [44]. Insomnia diagnostic criteria include trouble falling asleep, trouble staying asleep, or waking up too early for ≥ 3 nights/week for ≥ 3 months duration [19]. Another common sleep disorder is OSA. OSA has an estimated prevalence between 3 and 17%, which is even greater among men and older age groups [45]. OSA prevalence has increased over time with the upward trend in obesity and poses an increased risk for developing CVD [46]. This disorder is characterized by changes in sleep-related breathing that reduce or block airflow in the upper airways during sleep [47]. Early diagnosis and treatment may help reduce risk of comorbidities that are associated with unmanaged OSA, including hypertension [48]. Additionally, RLS—also known as Willis-Ekbom Disease—affects a significant share of the population, with estimates ranging from 3 to 11% [49]. Given the prevalence of these disorders and their impact on CVD-related outcomes, it is critical to consider the inclusion of screeners for insomnia, OSA, and RLS in sleep and cardiovascular research.

#### Insomnia Severity Index

The Insomnia Severity Index (ISI) is the most widely used clinical questionnaire for insomnia screening. It assesses sleep onset, sleep maintenance, early morning awakenings, satisfaction with sleep patterns, interference with daily functioning, and degree of concern about sleep problems [50]. The ISI items coincide with both the DSM (version IV) and International Classification of Sleep Disorders (10th edition) diagnostic criteria for insomnia [51]. The ISI internal consistency had a Cronbach’s α score of 0.92, a sensitivity of 82.4%, and specificity of 83.1% for detecting clinical insomnia in a primary care sample (*n* = 410) [51]. The ISI is also sensitive to change, making it an ideal measure for both behavioral and pharmacologic clinical trials of insomnia treatment [52]. ISI scores range from 0 to 28, with 7, 14, 21, and 28 marking the upper bounds of no clinically significant insomnia, subthreshold (mild) insomnia, moderate clinical insomnia, and severe clinical insomnia. In other words, a score of 15 or higher is often used as a threshold for clinically meaningful insomnia [50].

#### PROMIS Sleep Disturbance and Sleep-Related Impairment Short Forms

The National Institutes of Health developed the Patient-Reported Outcomes Measurement Information System (PROMIS) program using item-response theory with the goal of creating forms that more accurately measure numerous self-report constructs of interest than previously available questionnaires [53]. There are two sleep-related PROMIS measures, the Sleep Disturbance form and the Sleep-Related Impairment form. These forms are 8-item questionnaires with Likert responses ranging from one to five. There are adult and pediatric versions of both forms. Raw scores are converted into T-Scores with higher scores indicating greater sleep disturbance.

#### STOP-Bang Questionnaire

The STOP-Bang questionnaire was designed specifically to meet the need for a quick and easy-to-score screening tool for OSA [54]. The name of this questionnaire is an acronym that relates to common OSA features: snoring, tiredness, observed apnea, elevated BP, elevated body mass index, increased age, greater neck circumference, and male sex [54]. STOP-Bang scores range from 0 to 8 with 3–4 indicating an intermediate risk of OSA, while 5 and higher indicates high risk of OSA. The STOP-Bang questionnaire was created for adults over the age of 18 and has been validated against polysomnography (PSG), the objective gold standard for diagnosing OSA [55]. In a meta-analysis that examined data from over 17 studies and included nearly 10,000 people, the sensitivity for this questionnaire to correctly identify someone with risk of moderate-to-severe and severe OSA was at 94% and 96%, respectively [55].

#### Berlin Questionnaire

The Berlin Questionnaire was also created to screen for OSA [56]. The Berlin Questionnaire has 11 items that cover three sub-categories: snoring, daytime sleepiness, and history of hypertension [57]. If two or more sub-categories are endorsed, there is high risk for OSA. The Berlin Questionnaire has a high internal consistency (Cronbach correlations of 0.86 to 0.92) and has been validated against portable sleep monitoring machines such as PSG [56].

#### Cambridge-Hopkins Diagnostic Questionnaire

The Cambridge-Hopkins Diagnostic Questionnaire is an 11-item self-report measure designed to categorize people into “RLS,” “non-RLS,” and “possible RLS.” It consists of two core features of RLS and nine other, similar symptoms that are used in the differential diagnosis of RLS. It demonstrated 87.2% sensitivity and 94.4% specificity for diagnosing RLS [58].

#### International RLS Rating Scale

The International RLS Rating Scale is a 10-item self-report measure designed to assess the severity of RLS symptoms over the past week among those with an established RLS diagnosis. Its Likert responses range from 0 to 4. Examples of questions are: “Overall, how would you rate the RLS discomfort in your legs or arms?” and “How severe was your sleep disturbance due to your RLS symptoms?” [59] Scores range from 0 to 40, with 0–10 indicating mild RLS, 11–20 indicating moderating RLS, 21–30 indicating severe RLS, and 31–40 indicating very severe RLS.

#### Epworth Sleepiness Scale

Excessive daytime sleepiness is defined as difficulty maintaining alertness and wakefulness during the wake phase of the 24-hour sleep-wake cycle [19]. Some symptoms that correlate with excessive daytime sleepiness include trouble staying awake, trouble focusing, difficulty making decisions, and memory problems [60]. Excessive daytime sleepiness is one of the most prevalent symptoms of someone with an underlying sleep disorder such as OSA, narcolepsy, or idiopathic hypersomnia [61].

The ESS is used to identify excessive daytime sleepiness in both clinical and research settings [52], and it offers a standardized and cost-effective way to measure sleepiness in people who may have a sleep disorder [19]. This questionnaire asks about the likelihood of falling asleep during normal daily activities (e.g., watching television, driving, reading) to distinguish between sleepiness and fatigue [52]. Each daily activity has been chosen based on differing levels of “somnoficity,” a term referring to posture, activity, and the likelihood of that situation resulting in dozing [52]. Scores range from 0 to 24, with 0–5 indicating lower normal sleepiness, 6–10 indicating higher normal sleepiness, 11–12 indicating mild excessive daytime sleepiness, 13–15 indicating moderate excessive daytime sleepiness, and 16–26 indicating severe excessive daytime sleepiness.

#### Karolinska Sleepiness Scale

The Karolinska Sleepiness Scale was developed as a one-dimensional one-question sleepiness scale that is sensitive to change, as it asks about the 10 minutes prior to completing the questionnaire [62]. Respondents rate their current sleepiness on a 9-point Likert scale. A score of 7 (“sleepy, but no effort to stay awake”) or higher may indicate impaired performance. It has been widely used in studies about shift work, jet lag, driving abilities, attention, and performance [63]. Since it measures state rather than trait sleepiness, it is not used in clinical settings [62]. A study involving 16 female participants between 33 and 43 years of age required them to complete the Karolinska Sleepiness Scale eight times daily for three consecutive days [63]. There was a moderate correlation (*r* = 0.56) between higher Karolinska Sleepiness Scale and increased reaction times, number of lapses, alpha and theta power density, and alpha attenuation coefficients [63]. The Karolinska Sleepiness Scale results were also compared with other behavioral variables and electroencephalogram (EEG), and had high validity for measuring sleepiness [63]. The Karolinska Sleepiness Scale has also been validated in a Spanish-speaking Colombian population [64].

#### Stanford Sleepiness Scale

The Stanford Sleepiness Scale, like the Karolinska Sleepiness Scale, evaluates one’s state sleepiness at the time of measure administration with a 7-point Likert scale [65]. The Stanford Sleepiness Scale also includes just one item, asking the respondent to rate their sleepiness at that specific time [66]. Thus, it is best used when repeated throughout a research study or over the course of treatment to track changes [65]. Scores of 5–7 are generally considered functionally impaired. To evaluate the Stanford Sleepiness Scale, five college students were given the questionnaire for five consecutive days and were sleep deprived on the fifth day [65]. After sleep deprivation, scores for the Stanford Sleepiness Scale increased significantly, which correlated with predicted levels of sleepiness [66]. The Stanford Sleepiness Scale was moderately correlated to sleep deprivation among college students (*r* = 0.474) [66].

#### Functional Outcomes of Sleep

The Functional Outcomes of Sleep Questionnaire (FOSQ) is used to determine the effects of fatigue on daytime activities and quality of life among individuals with a sleep disorder [67]. It has both long (30 items) and short (10 items) versions [68]. The short version is specifically for clinical use as completing and scoring are less time consuming [69]. The FOSQ evaluates five categories: activity level, vigilance, intimacy and sexual relationships, general productivity, and social outcomes [52]. FOSQ-30 and FOSQ-10 scores range from 5 to 20, with 17.9 or lower indicating functional impairment. The FOSQ-10 covers the same domains as the FOSQ-30 but in abbreviated form. It has the same score range and clinical interpretation as the FOSQ-30. The FOSQ-30 has high internal reliability (α = 0.95) and test-retest reliability (*r* = 0.90), demonstrating consistency in respondent reporting over time [70].

#### Dysfunctional Beliefs and Attitudes about Sleep Scale

When conducting sleep research, it is crucial to understand the beliefs a patient may hold about sleep, as they can perpetuate sleep disturbance. The Dysfunctional Beliefs and Attitudes about Sleep Scale (DBAS) is a 30-item self-report questionnaire designed to assess a person’s unhelpful sleep-related cognitions. The score is calculated as a mean of the item scores. If the mean score is greater than or equal to 3.8, this indicates elevated dysfunctional beliefs about sleep, There is also an abbreviated, 16-item version of the measure (DBAS-16), which has demonstrated adequate internal consistency (α = 0.77–0.79) and temporal stability (*r* = 0.83) [71].

### Objective Sleep Measures

Objective sleep measures are often perceived as a more rigorous and accurate means of measuring sleep. However, we and others contend that subjective and objective measures should be viewed as separate constructs of equal value as they are sometimes discordant, particularly among older adults and in certain clinical populations [72–75]. For example, studies report that 36–39% of individuals who report having insomnia do not have poor quality sleep according to TST, SE, and WASO measured with actigraphy [76]. Thus, research studies often benefit from collecting both subjective and objective measures of sleep rather than prioritizing one over the other. The primary ways to obtain an objective measurement of sleep are with actigraphy or PSG. Both objective and subjective sleep measures are summarized in Table 1.

### Actigraphy

An actigraph is a wrist-worn device that uses accelerometry (typically tri-axial accelerometry) to measure movement. As such, actigraphy does not directly measure sleep; rather, it distinguishes between periods of sleep and wakefulness based on movement. While consumer-grade wearables have improved in their ability to differentiate sleep from wakefulness and have shown validity for some objective sleep parameters compared with PSG, research-grade devices remain the recommended tools for objective sleep research [77–83]. Actigraphy is useful in the assessment of TST and SE, as well as circadian rhythm sleep-wake disorders. There are numerous advantages to using actigraphy (Table 1): are non-invasive and well tolerated; allow longitudinal assessment of sleep and circadian rhythms for weeks to months (depending upon device settings and battery life); allow measurement of sleep in the home environment for better generalizability; enable collection of data on light exposure in some devices. It is recommended that actigraphy data be collected along with sleep diaries for a minimum of seven days and preferably for 14 days to determine differences between weekdays and weekends. For additional details beyond what is summarized below, refer to the American Academy of Sleep Medicine or Society of Behavioral Sleep Medicine clinical and research guidelines on the use of actigraphy [77, 84, 85].

**Fig. 1:**
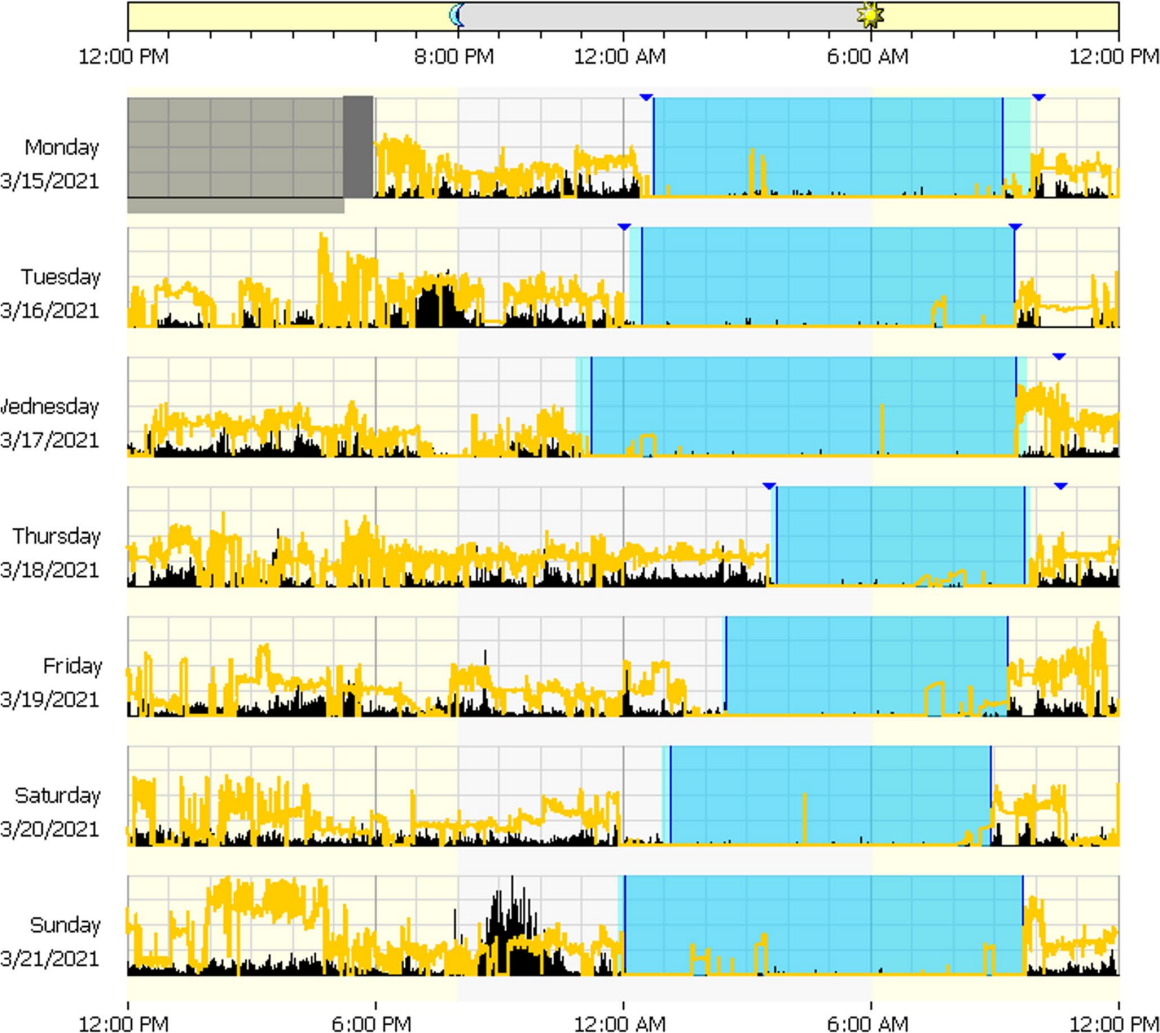
An example actogram. This actogram presents data obtained over 7 days. Each row of data is a 24-hour period. The x-axis begins at 12:00 pm, the midpoint of the axis is at 12:00 am (midnight), and the x-axis ends at 12:00 pm the next day. The blue box is the identified sleep period. This person has a regular wake time (9:00 am) and appeared to have shorter sleep periods than normal on 03/18/2021 and 03/19/2021

Once an individual returns the actigraphy device, the data are downloaded and proprietary software manually scores the data to identify sleep periods and produce a report of variables, including SOL, TST, WASO, SE, and a sleep fragmentation index, among others. The sleep diary is used to make any necessary modifications to the software scoring. For example, it is not unusual for the software algorithm to identify time spent in bed watching television as sleep. In this case, sleep onset may be manually moved within the software to be more consistent with the individual’s sleep diary report of sleep onset time. An example actogram is presented in Fig. 1.

**Fig. 2:**
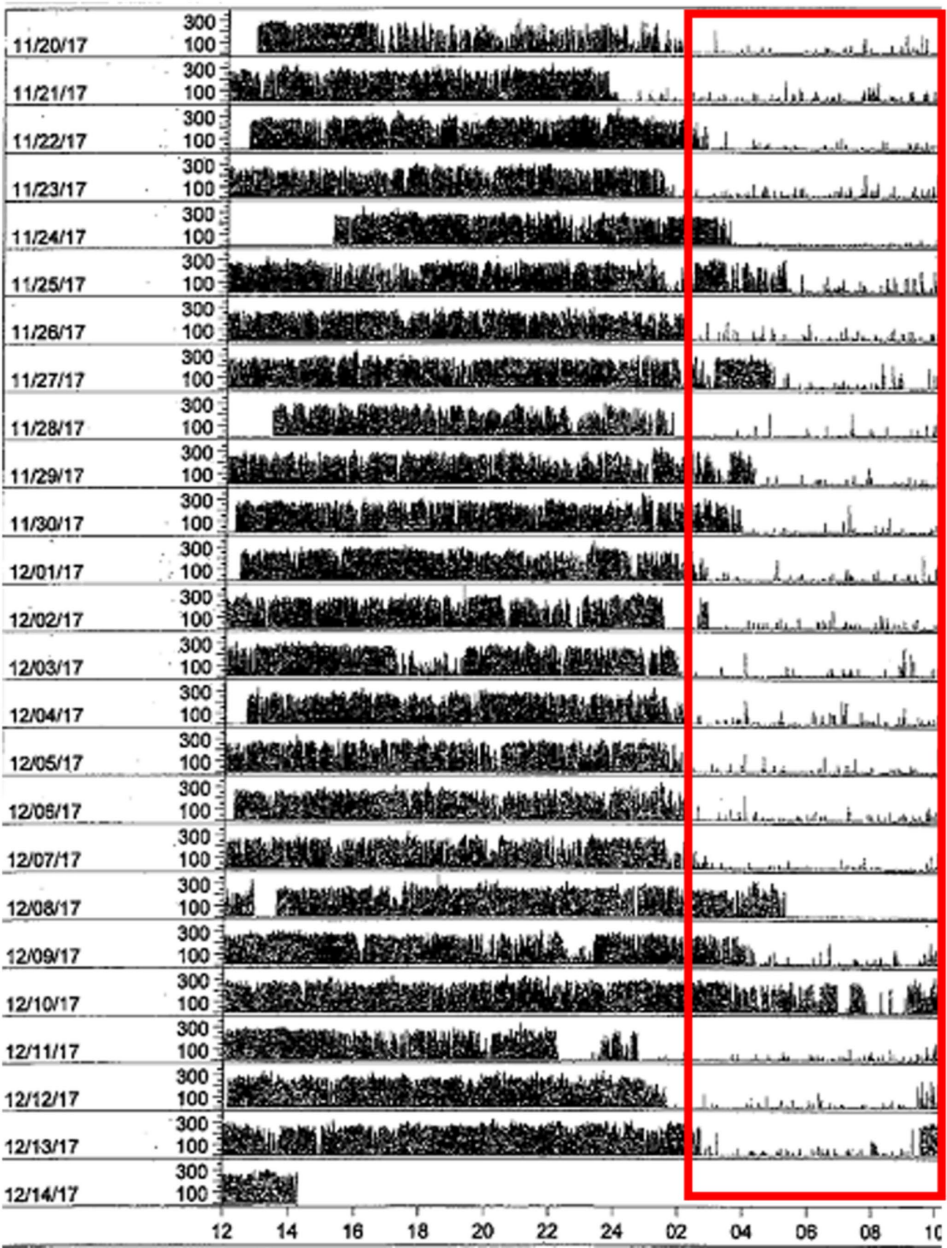
Actogram from an individual with delayed sleep-wake phase disorder. This actogram presents data obtained over 24 days in an individual with delayed sleep-wake phase disorder. Each row of data is a 24-hour period. The x-axis begins at 12:00 pm, the midpoint of the axis is at 12:00 am (midnight), and the x-axis ends at 12:00 pm the next day. The red box is the identified sleep period. Bedtime was rarely before 2:00 am and wake time was generally not until 12:00 pm. On 12/10/2017, the individual did not appear to sleep as evidenced by robust activity throughout the 24-hour period, including during the habitual sleep period

In addition to the output provided on the sleep parameters described above, visual inspection of the actogram can reveal sleep/wake behaviors consistent with an underlying circadian rhythm sleep-wake disorder. An individual with delayed sleep-wake phase disorder is depicted on an actogram (Fig. 2), wherein sleep onset occurs between 2:00 am – 4:00 am and sleep offset (wake time) is generally 12:00 pm. Algorithms also allow for the estimation of circadian parameters, including parametric (e.g., acrophase, amplitude, mesor, pseudo-F statistic) and non-parametric (interdaily stability and intradaily variability) rest-activity rhythms. Recently, an algorithm was developed to predict DLMO from actigraphy data obtained in a sample of shift workers [86].

### Polysomnography

PSG is considered the gold-standard for objective sleep assessment and involves measuring multiple physiological parameters to identify and stage sleep. Additionally, PSG is required in the diagnosis of many sleep disorders, including sleep disordered breathing (e.g., OSA or central sleep apnea), periodic limb movement disorder, and parasomnias (e.g., REM sleep behavior disorder). Of note, PSG is not indicated in the diagnosis of insomnia [87, 88], in part because insomnia is a diagnosis based on self-report [89, 90]. Though PSG is considered the gold standard for objective sleep assessment and is the only means of staging sleep (i.e., stages N1, N2, N3, and REM), it is limited by its cost and invasiveness. Studies will often collect two nights of PSG because of the “first night effect,” which refers to a departure from habitual sleep quality or quantity due to being outside of the home environment [75]. PSG may be impractical when measuring more than a few nights of sleep, in which case research studies typically employ sleep diaries and/or actigraphy.

PSG channels typically include electroencephalography (EEG), electrooculography (EOG), electromyography (EMG), electrocardiography (ECG), a nasal cannula for nasal pressure, respiratory effort belts, and pulse oximetry. EEG is used to identify sleep onset and stage sleep as N1, N2, or N3. EEG is used in combination with EOG and EMG to identify REM sleep. PSG is ideal if a precise measurement of sleep onset or sleep staging is needed, or when EEG frequencies are being assessed. In these cases, a basic montage comprised of EEG, EOG, and EMG is sufficient. Sleep parameters obtained from PSG include many of the same variables collected by actigraphy (e.g., SOL, TST, WASO, and SE), as well as number of EEG arousals and time spent in N1, N2, N3 and REM sleep.

When additional parameters beyond sleep staging are needed, additional leads are added. For example, if an assessment of sleep disordered breathing is needed, respiratory channels (i.e., nasal pressure, respiratory effort, and pulse oximetry) can be added to the montage. If movement during sleep is being assessed (e.g., periodic limb movement disorder or parasomnias), EMG leads can be added to the arms and/or legs. Additional sleep parameters may include number of apnea and/or hypopnea, calculation of the apnea hypopnea index (AHI; number of apnea and hypopnea per hour), oxygen saturation, limb movements, and calculation of a limb movement index.

While PSG is most often performed in a clinic setting where the PSG equipment is installed and difficult to move, ambulatory PSG devices are available to measure common PSG channels in any location desired (type 2 devices). Additionally, home sleep testing devices are available to measure only the channels relevant for identifying sleep disordered breathing (type 3 devices). While type 3 devices do not offer the comprehensive assessment afforded by in-lab PSG or type 2 devices, they are a more rigorous assessment of sleep disordered breathing compared with the questionnaires designed to assess risk for sleep disordered breathing (e.g., STOP-BANG or Berlin questionnaires).

Objective sleep measures are valuable tools to provide sleep data unbiased by an individual’s subjective impression of their sleep. PSG is the gold standard means of identifying and staging sleep, and actigraphy is valuable in the longitudinal assessment of sleep parameters. Actigraphy can also provide an estimate of circadian parameters, however the gold standard methods for assessing markers of circadian rhythms in humans are discussed in the section below.

### Circadian Research Methods

Circadian rhythmicity is most effectively assessed in a laboratory setting where there is enhanced control of environmental and behavioral factors (e.g., light, meal timing, physical activity) that mask and influence circadian rhythms in daily life [91]. Two commonly used laboratory-based research methods to determine circadian phase, amplitude, and/or period in humans are the forced desynchrony and constant routine protocols [92–94]. While an estimation of circadian amplitude can be derived from data collected during either the forced desynchrony or constant routine protocol, the forced desynchrony protocol is the gold standard method to measure circadian period and is conducted over a period of one to four weeks [93, 95]. During this protocol, a fixed sleep-wake schedule and non-24-hour day (e.g. 20-, 28-, 42.5-hour) are imposed on participants to desynchronize the circadian pacemaker and sleep-wake behaviors, during which all time cues are removed from the environment, such as clocks and phones [96].

The constant routine protocol is considered the gold standard method to measure circadian phase by eliminating external entrainment cues (e.g., light exposure) or evenly distributing those that cannot be eliminated (e.g., meal timing) to observe endogenous circadian rhythms. During the protocol, participants remain in a semi-recumbent posture in constant dim light (< 5–10 lx) and temperature, consume iso-caloric snacks in evenly timed intervals (e.g., one or two hours), and maintain constant wakefulness. Participants must remain in constant conditions for more than 24 hours, allowing at least five to six hours for the masking effect of external entrainment cues to dissipate before data collection. Circadian markers, including melatonin, cortisol, and core body temperature, are repeatedly measured during the constant routine protocol to detect rhythmicity at different intervals discussed below. Using the constant routine protocol, the oscillation of this waveform can be assessed to separate a rhythmic pattern from periodic behavior [92]. A constant routine protocol may also be performed before and/or after a forced desynchrony protocol to assess endogenous circadian phase [97]. When designing a human circadian research study, it’s critical to consider available resources, how the controlled environment may affect cardiovascular functioning, and which circadian marker(s) will best address the research question.

### Common Circadian Biomarkers

#### Melatonin

Melatonin, a hormone secreted by the pineal gland, is involved in regulation of the sleep-wake cycle and is considered a precise marker of the human circadian clock [93, 98]. Its production peaks approximately two hours before sleep onset and falls gradually throughout the night with a sharp fall just before awakening; however, melatonin production is suppressed in the presence of light exposure, especially short wavelength light between 446 and 477 nanometers (i.e., blue light) [98–100]. There are different markers to indicate melatonin timing (e.g., melatonin onset or offset, fitted melatonin peak); however, melatonin onset is the most common [101]. DLMO assessment is a widely used method of measuring circadian phase and requires sample collection every 30–60 minutes in dim lighting [102, 103]. Melatonin can be reliably measured in saliva, blood, and urine, although saliva collection is more cost-effective and could be completed by research participants in their home [93, 104–107]. Not only are there inter-individual differences in melatonin peak, but there are also differences in melatonin levels based on sample type, which necessitates different thresholds when quantifying and interpreting endogenous melatonin levels [108–110]. The threshold to determine salivary DLMO is 3–4 pg/mL or two standard deviations above daytime melatonin levels while the plasma DLMO threshold is typically 10 pg/mL [104, 106]. The standard deviation approach is especially useful when individuals have low melatonin levels (not exceeding 3–4 pg/mL) and thus, a more subtle rhythm.

Once samples are collected, a radioimmunoassay or enzyme-linked immunosorbent assay (ELISA) is performed to detect and interpolate melatonin concentration. In our lab’s most recent study aiming to assess circadian contribution to BP variation in Black adults (manuscript in preparation), we collected saliva hourly and measured salivary melatonin levels using the Buhlmann Laboratories radioimmunoassay kit (RK-DSM2). The higher accuracy and sensitivity of radioimmunoassay compared to ELISA was preferential in this case given racial differences in melatonin levels [111]. A critical analysis of melatonin assaying and commercially available kits has been recently published and can serve as a guide for sample collection, analyses, and interpretation [112].

#### Core Body Temperature

Core body temperature exhibits a robust circadian rhythm, typically reaching a peak in the early evening and a trough in the early morning, though this may differ for those with extreme morning or extreme evening chronotypes, as well as shift workers [113, 114]. We use clock time in this description for ease of visualization but it is more precise to talk about circadian rhythms in reference to an individual’s biological or internal timing, also called circadian phase. The rhythm of core body temperature mirrors the rhythm of BP, with both peaking just prior to sleep onset and reaching a nadir a few hours before habitual awakening in most individuals [115–117]. The parallel rhythm between core body temperature and BP may be driven by the central circadian clock. Notably, some individuals show variation in the typical 24-hour BP pattern, like those with OSA and RLS. Those with OSA often show non-dipping or even reverse nighttime dipping, or elevated BP throughout the day. Those with RLS may also show higher BP during wake periods, as well as BP surges after limb movements [118, 119]. Core body temperature has often been measured via rectal thermometer, including a study investigating circadian rhythmicity of BP, which allows for consistent data collection for an extended period of time [93, 116]. This approach may be a barrier to enrollment for some individuals and can also produce artificially high readings [120]. Alternatively, there are ingestible pills and monitors that transmit core body temperature via Bluetooth technology while the sensor is in the research participants’ gastrointestinal tract [121]. It is common practice to use the lowest recorded core body temperature as a circadian phase reference marker for standardization and alignment of data (e.g., temperature, blood pressure) by each participant’s own circadian phase prior to data analysis; use of clock time for data alignment is incorrect and a circadian phase marker as a reference for biological time is required when conducting circadian rhythm research [122].

#### Cortisol

Using an individual’s wake time as the circadian phase marker or start of the circadian rhythm cycle, a typical person’s cortisol secretion peaks early in a person’s day, usually 20 to 30 minutes after waking up, and the lowest levels are observed in the middle of sleep, with short bursts of cortisol secretion over the course of a waking period [123, 124]. This steroid hormone plays a role in circadian synchronization by acting as a secondary messenger between the central and peripheral clocks [124]. Similar to melatonin, cortisol can also be reliably measured in saliva, blood, and urine and quantified with immunoassays [125]. In many experimental procedures including the constant routine protocol, cortisol levels are assayed in salivary samples. Salivary glands release the unbound plasma of cortisol into the mouth, which can be obtained through this noninvasive and ethically safe method [123]. Participants can be tested over a full 24-hour day and sample collection is recommended every 20–30 min [103].

## Statistical Analyses

Statistical methods used to assess sleep and circadian rhythms in humans can be complex and are not often used in other cardiovascular-related studies. Selecting the appropriate statistical test is vital to drawing accurate conclusions. The statistical approach most often used in circadian studies typically involves detecting rhythmicity, characterizing the rhythm, if present, and comparing key parameters of rhythms. The sections below are intended to serve as an overview of statistical methods used in human circadian research studies and are summarized in Table 2. More detailed information about statistical tests and considerations for analyzing time series data can be found in published articles [126–128]. There are also several statistical software packages designed to analyze time series data that are publicly available online. Consulting with an experienced statistician is highly recommended to determine the most appropriate statistical procedures.

**Table 2:** Common statistical tests to detect and characterize circadian rhythms

Test	Method Used to Detect Rhythmicity	Key Parameter Estimation	Example of Applications in Cardiovascular Research	References
Fourier Analysis	Applies sine and cosine waves with varying frequencies to time series data to detect rhythmicity	Period	Circadian and ultradian rhythmicity in pediatric populations [157, 158]	[135, 136]
Cosinor Analysis	Fits a cosine wave function to time series data using the least squares method	Phase, mesor, and amplitude	Circadian rhythms in human blood pressure [116]	[140]
Rayleigh Test	Determines the directionality in a circular distribution of data	Period, phase	Pattern of onset of myocardial infarctions in young people [151]	[150]

### Data Analysis Considerations

Before conducting analyses, it is important to visually inspect the raw data as a function of time to prevent inappropriate use of statistical tests. This will also provide some initial insights on rhythmicity (e.g., number of peaks), shape of the data and increasing or decreasing trends [129]. Scatterplots and dot plots can help to determine outliers, erroneous values, and sample distribution that may not be apparent using descriptive statistics alone. Researchers are encouraged to plan how to treat outlier data prior to data collection with careful consideration of the expected distribution and relevant biological knowledge [126]. Circadian data is internal or biological, rather than external, time-dependent, and both sampling rate and duration of data collection are important to consider during study design and analysis preparation. For example, mean ambulatory BP is most accurate when read in less frequent intervals (e.g., 30–60 minutes) across multiple consecutive 24-hour cycles (e.g., 48 hours) [92, 130, 131]. Missing data due to sample collection issues may require a change in analytic approach, or exclusion of participants with missing data over a specific threshold (e.g., ≥ 30% missed BP readings), with careful consideration of when during the 24-hour period data are missing [132]. Interpolation of biological data is not recommended, particularly for time-series analyses (e.g. cosinor), which remain valid despite irregular sampling intervals and incomplete datasets [128]. Instead, missing data should be accommodated through analyses within equal time units (e.g., hourly) [133].When the number of observations is large, leaving invalid points as missing generally minimizes bias, and analyses can proceed with methods that accommodate irregular or unbalanced observations (e.g., mixed-effect models) [134]. Given the repeated measurements within individuals, mixed-effects modeling may be beneficial to account for both intra- and inter-individual changes, as well as changes due to the experimental condition [127].

### Common Statistical Analyses to Detect Circadian Rhythmicity

#### Fourier Analysis

Fourier analysis or “spectral analysis” is a statistical test that applies sine and cosine waves with varying frequencies to time series data to detect rhythmicity [135, 136]. This statistical analysis has been applied to cardiovascular research for several years, especially regarding BP patterns [137–139]. Methods such as the Fast Fourier Transform can be used to parse out different frequencies or periods in the data [127].

#### Cosinor Analysis

Cosinor analysis is a method of detecting rhythmicity by fitting time series data to a cosine wave function of a pre-specified period (e.g., 24-hour alone or with additional harmonics) using the least squares method [128, 140]. Several adaptations have been developed to produce additional information, such as calculating parameter estimations and handling non-linear data among other features [141–144]. Cosinor analysis has been used to determine circadian contribution to BP and detect rhythmicity [116]. Rhythmicity can be detected using a zero-amplitude test, which tests the null hypothesis that the amplitude is zero; an amplitude statistically greater than zero implies 24-hour rhythmicity in the time series data [128, 129, 145]. Special attention should be given to the shape of the data prior to performing cosinor analysis, which underscores the importance of visual inspection prior to performing analyses. Attempting to fit the data to a cosine function when the shape of the data is incongruent will produce results that are not truly meaningful [127, 146]. An alternative to using cosinor analysis that attempts to fit time series data to a function is to opt for a nonparametric test such as a smoothing spline, which does not assume a function [127, 146]. Similarly, alternative approaches may be required if the variable of interest exhibits non-stationarity (e.g., a linear drift) due to either a biological process, methodological artifact, or behavioral influence. This could be addressed by adding a linear component to the existing sinusoidal model. Further, a multi-component cosinor model is likely needed to model time series data when the waveform exhibits a non-sinusoidal pattern (e.g., bimodal rhythm) [128, 129]. For example, 2- or 3-component cosinor models (24-hour, 12-hour harmonic, and linear components) have been used to detect and characterize the circadian rhythmicity of BP [116, 147–149]. Elaboration on cosinor analyses can be found in a review by Cornelissen [129].

#### Rayleigh Test

The Rayleigh test can be applied when a variable of interest does not occur in even intervals and provides information about the directionality of the distribution of data collected [150]. For example, this procedure has been used to characterize the pattern of symptom onset in young patients who have had acute myocardial infarctions. Using the Rayleigh test, Rallidis and colleagues identified onset of ST-segment elevation acute myocardial infarction (AMI) symptoms (e.g., atypical chest pain, gastrointestinal problems, weakness) peaked during the morning in young patients with AMI [151]. This study exemplifies the benefits of using circadian analyses to enhance identification and subsequent treatment of cardiovascular problems. With the improvement of and widespread use of modern-day wearables, heart rate may become a more accessible circadian biomarker than DLMO.

### Key Circadian Rhythmicity Parameters

To conduct a cosinor analysis and estimate key parameters, one must identify a circadian phase marker and align the data, as discussed above. Subsequently, circadian rhythmicity can be tested using cosinor analysis. It is customary to calculate key parameter estimates only after circadian rhythmicity has been detected in a variable of interest. After circadian rhythmicity is confirmed, the following four key parameters of circadian rhythmicity can be estimated: acrophase, MESOR, period, and amplitude. Acrophase is one of the key parameters derived from the cosinor analysis that refers to the peak (i.e., highest point) of the rhythm. Conversely, the bathyphase refers to the trough (i.e., lowest point) of the rhythm [128]. More broadly, circadian phase refers to the timing of an event in relation to a reference marker (typically the onset of an event). Longitudinal data collection introduces the variability within the reference marker from day to day; therefore, phase estimations should be based on onset over several days. In this case, linear regression using daily onsets would be recommended to estimate phase [128].

MESOR (Midline Estimating Statistic of Rhythm) is the rhythm-adjusted mean of time series data, derived from a cosinor model. It is the central value around which the oscillation occurs. When data points are equidistant and cover an integer number of cycles, the MESOR is equal to the arithmetic mean of all data points. However, when data points are not equidistant and/or do not cover an integer number of cycles, MESOR deviates from the arithmetic mean and often provides an accurate and precise estimate of the central tendency of time series data [128].

“Period” refers to the duration of a complete circadian rhythm. For example, the circadian clock in humans typically has a circadian period of 24.18 hours that entrains to a 24-hour cycle because of environmental cues [93]. In the past, circadian period has been derived from actograms; however, this method has limitations, including subjectivity and relying on a small portion of time series data. Other methods, including Fourier analysis, Lomb-Scargle periodogram, or linear and non-linear cosinor analysis, have been used to determine circadian period with varying degrees of sensitivity to detecting multiple circadian components with distinctly different periods [128].

Amplitude provides information about the intensity of a rhythm, but more formally, is the term used to describe the distance between the midline (MESOR) and the highest (peak/acrophase) or lowest (trough/bathyphase) point in an oscillation. It can also be calculated simply as half the value from peak to trough, although this method is more susceptible to outliers. Alternatively, circadian amplitude is calculated by parameter estimation during cosinor analysis [128]. Some studies have investigated circadian amplitude of BP in the past and associated higher amplitude with increased cardiovascular morbidity and mortality; however, the majority of the literature supports associations between low systolic BP amplitude and increased cardiovascular morbidity and mortality [152–154].

## Conclusion

As research progresses, sleep and circadian rhythms are further implicated in a variety of health outcomes including CVD events. As such, it is imperative to understand the measures of both sleep and circadian rhythms when studying mechanisms of CVD development. We have summarized the most widely used measures of sleep. We recommend the use of both objective and subjective assessments of sleep given their potentially discrepant but complementary data [155]. Circadian rhythms research requires rigorous methods to produce accurate results free of masking effects; our review highlighted the two methods that allow measurement of circadian rhythms (forced desynchrony and constant routine protocols) and identified the situations in which they should be used.

Sleep is a critical component of cardiovascular health and thus, should be considered both unidimensionally and multidimensionally to better understand its impact on long-term BP and CVD risk. Unidimensional approaches can clarify discrete pathways between sleep facets and CVD outcomes, while multidimensional approaches (e.g., considering the combined influence of sleep duration, quality, and chronotype) can better capture the complex pathways between sleep and CVD. This review equips hypertension researchers to address major gaps in CVD etiology and treatment. When designing a research study, it is crucial to consider which assessments will effectively measure the desired aspect of sleep or circadian rhythms. We have provided an overview and offered guidance on selecting appropriate measures based on the research question. Incorporating sleep and circadian measures into cardiovascular health research will expand our understanding of the links between them [3, 156].

## Data Availability

No datasets were generated or analysed during the current study.

## Glossary of Terms

AHI: Apnea Hypopnea Index
BP: Blood pressure
CVD: Cardiovascular disease
DBAS: Dysfunctional Beliefs and Attitudes About Sleep Scale
DLMO: Dim light melatonin onset
DSM-5: Diagnostic and Statistical Manual V
ECG: Electrocardiography
EEG: Electroencephalogram
ELISA: Enzyme-linked immunosorbent assay
EMG: Electromyography
FOSQ: Functional Outcomes of Sleep Questionnaire
ISI: Insomnia Severity Index
MCTQ: Munich Chronotype Questionnaire
MEQ: Morningness-Eveningness Questionnaire
OSA: Obstructive sleep apnea
PROMIS: Patient-Reported Outcomes Measurement Information System
PSG: Polysomnography
PSQI: Pittsburgh Sleep Quality Index
REM: Rapid eye movement
RLS: Restless Legs Syndrome
RU-SATED: Regularity, Uninterrupted Sleep, Satisfaction, Alertness, Timing, Efficiency, and Duration
SATED: Satisfaction, Alertness, Timing, Efficiency, and Duration
SCISD-R: Structured Clinical Interview for Sleep Disorders-Revised
SE: Sleep efficiency
SOL: Sleep onset latency
TIB: Time in bed
TST: Total sleep time
WASO: Wake after sleep onset
